# Beyond adult models: *Tribolium castaneum* larval timekeeping reveals unexpected robustness and insights into circadian clock

**DOI:** 10.1111/1744-7917.13437

**Published:** 2024-08-09

**Authors:** Miriam Benita, Ariel Menahem, Animesha Rath, Inon Scharf, Daphna Gottlieb

**Affiliations:** ^1^ Department of Food Science, Institute of Post‐Harvest and Food Science The Volcani Center Rishon LeZion Israel; ^2^ George S Wise Faculty of Life Sciences School of Zoology Tel Aviv University Tel Aviv Israel

**Keywords:** conditioned flour, development, 4,8‐dimethyldecanal, endogenic clock, quinone, *T. castaneum*

## Abstract

Circadian rhythms are self‐sustained endogenous oscillations that are found in all living organisms. In insects, circadian rhythms control a wide variety of behavioral and physiological processes, including feeding, locomotion, mating, and metabolism. While the role of circadian rhythms in adult insects is well‐understood, it is largely unexplored in larvae. This study investigates the potential for larval synchronized activity in the red flour beetle (*Tribolium castaneum*), a species exhibiting solitary and aggregation phases. We hypothesized that, similar to adults, larvae would exhibit a daily activity pattern governed by an endogenous circadian clock. We further predicted that the transition between the solitary and gregarious phases extends to unique temporal activity patterns. Our results revealed unique timekeeper gene expression in larvae, leading to a distinct daily rhythm characterized by nocturnal activity. Cues indicating on potential cannibalism did not change daily activity peak. However, the absence of these cues significantly reduced the proportion of rhythmic larvae and led to higher variation in peak activity, highlighting the crucial role of social interactions in shaping their rhythmicity. This study sheds light on the evolution and function of larval synchronization in group‐living insects, offering novel insights into this complex behavior.

## Graphical abstract

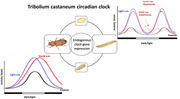

## Introduction

Organisms face environmental challenges resulting from cyclic variations in biotic and abiotic factors, such as light, temperature, humidity, predators, reproductive potential, and food availability. These environmental changes are hypothesized to have driven the evolution of highly conserved biological timekeeping systems (Panda *et al.*, 2002; Morgan, 2004). Almost all organisms have an internal timekeeper system, a circadian clock, entrained by external stimuli that helps them to regulate a variety of physiological and behavioral processes, including feeding, reproduction (Panda *et al.*, 2002) and hatching time (Minis & Pittendrigh, 1968). In group‐living organisms, daily temporal activity synchronizes with various external cues, including food availability, predator presence, and interactions with group members. Similar to synchronizing temporal activity with the external environment, temporal synchronization with the social environment is fundamental and is evident in a large range of insects (Gottlieb, 2019). Examples exist from the collective bioluminescent flashing by lampyrid beetles or fireflies (Ramírez‐Ávila *et al.*, 2018) to the synchronized bee hive (Siehler *et al.*, 2021) and is best known in some acoustic orthopterans (Greenfield & Merker, 2023). Despite increasing research on group‐living insects, temporal synchronization primarily focuses on adults, neglecting the juvenile stage (except for some lepidopteran studies Niepoth *et al.*, 2018).

The question of whether larvae synchronize their temporal daily activity remains largely unanswered. Larval synchronization patterns likely differ between social and solitary species. Larvae of social insects, like ants or bees, heavily depend on nest‐mate care (Boomsma, 2009) and are shielded from the external environment. Thus, larvae of social species with full parental care may have weaker daily rhythmicity (Fuchikawa *et al.*, 2016; Fujioka *et al.*, 2017; Fujioka *et al.*, 2019; Beer & Helfrich‐Förster, 2020). Conversely, in solitary species where larvae lack extensive adult care and face direct environmental pressures, synchronization between adults and larvae may be less apparent. However, potentially synchronized behavior might arise due to a common external stimulus. For example, in Lepidoptera, caterpillars of the day‐active butterfly *Danaus plexippus* and the night‐active moth *Heliothis virescens* do not exhibit diurnal or nocturnal activity similar to their adult counterparts. Instead, external forces, such as temperature and host plant, can lead to rhythms similar to adults rhythms in feeding and activity (Niepoth *et al.*, 2018).

The present study seeks to clarify how external and social rhythmic cues differentially contribute to the rhythmic behavior of larvae living in aggregations. We focus on the temporal locomotor daily activity of red flour beetle (*Tribolium castaneum*) larvae and compare to adults locomotor daily activity pattern. *Tribolium* beetles, major pests of stored food products (Srivastava & Subramanian, 2016), exhibit remarkable plasticity by switching between solitary and gregarious phases throughout their lifespan (Sokoloff, 1977). Adult female activity patterns strongly depend on social conditions. Notably, solitary females peak later in the light phase, while those in aggregations peak earlier, a shift suggested to reduce intermating probability within mixed‐species aggregations (Benita *et al.*, 2024). This shift is also observed in the species’ circadian molecular clock (Rath *et al.*, 2021). The circadian clock's core mechanism involves a roughly 24‐h transcriptional/translational negative feedback loop driven by oscillations of *Tcper* and *Tctim* transcripts and proteins (Li *et al.*, 2018). When PERIOD (PER) and TIMELESS (TIM) proteins reach critical cytoplasmic concentrations, they translocate to the nucleus and inhibit their own transcription. Rath *et al.* (2021) found that the peak of *Tcper* is in the photophase and *Tctim* is in the scotophase. Light and temperature cycles entrain the endogenous clock to a 24‐h period, while odor signals indicating a gregarious environment up‐regulate *Tctim* (Rath *et al.*, 2021). The present study seeks to elucidate the differential contributions of external and social rhythmic cues to the rhythmic behavior and circadian molecular clocks of larvae living in aggregations.

There are two major odor signals of communication between individuals within an aggregation. At low densities and ample food resources, aggregation pheromone (4,8‐dimethyldecanal, henceforward DMD) is primarily produced by males (Dissanayaka *et al.*, 2018). In overcrowded conditions, both males and females release benzoquinones (El‐Desouky *et al.*, 2018) into the flour, acting as antiaggregation pheromones and promoting larval cannibalism, dispersal or reduced aggregation behavior (Duehl *et al.*, 2011). Similar to adults, larvae display attraction to male‐produced aggregation pheromone and repulsion in response to benzoquinones (Mondal, 1985). Nonchemical signals can also indicate the population density. In densely aggregated populations, the intensity and frequency of vibrations is a reliable cue for individual organisms to assess population density (Ishihara *et al.*, 2021). As high vibrations lead to higher activity (Ishihara *et al.*, 2021), these cues can play a role in inducing distinct larvae daily activities.

The primary goal of this study was to assess larval daily temporal synchronization in group‐living insects and to compare it to the adults’ temporal synchronization documented in Rath *et al.* (2021) and in the current research. To quantify and properly compare adults and larvae temporal activity, we found it crucial to examine the same parameter, locomotion, in both stages. Our first prediction was that larvae, like adults, possess an endogenous clock. We further predicted a plastic peak activity time: larvae in the solitary stage, or those primarily exposed to aggregation pheromone, would exhibit daily activity patterns similar to adults. However, when facing a risk of cannibalism, that is, exposure to conditioned flour or close proximity to starved adults (Park *et al.*, 1965; Parsons *et al.*, 2013), we hypothesized that larvae would synchronize their activity to the social environment, switching to nocturnal activity to achieve temporal segregation.

## Materials and methods

### Insect rearing

*Tribolium castaneum* were obtained from a mill storehouse in the northern Galilee region of Israel. The beetles were lab‐reared for 10–15 generations on 250 g of white flour under 12 : 12 h light : dark (hereafter, LD) regime, 65% ± 5% relative humidity, and 25 °C. Population size and age were controlled by removing the eggs to a new rearing jar every two months and killing the adults and the larvae. The beetles, larvae and adults for the experiments were taken from a population of approximately 50 adult beetles per rearing jar and were at a similar age. While sex ratio was not controlled, we assume it is balanced. In all experiments, we estimated the activity of last instar larvae (4.5 ± 0.5 mm length). As a result, all experiments were limited to a short duration before the larvae pupated.

### Behavioral experiments

**Endogenous circadian clock**: 36 larvae were selected and measured, 4.5 ± 0.5 mm in size, and individually placed in a 24 well plate. The experiment began with a 24‐h period under a 12 : 12 LD cycle (light intensity: 625 lux) at room temperature. This was followed by 48 h of complete darkness, and a final 24‐h period under the 12 : 12 LD cycle. Throughout the experiment, the plates were placed under a DanioVision camera for continuous tracking the larval movement and activity (distance traveled per 10 min).

**Exposure to conditioned flour**: 48 larvae (4.5 ± 0.5 mm in length) were separately placed in wells, half of which received fresh flour and the other half conditioned flour. This conditioned flour, obtained from a 4‐month‐old overcrowded beetle stock jar (prepared specifically for this experiment), exposed the larvae to cues indicating a high‐risk cannibalism environment. The plate was then placed under DanioVision camera for three days with continuous tracking of larval movement and activity (distance travelled per 10 min) under room temperature and a 12‐h light (625 lux)/12‐h dark cycle.

**Exposure to DMD**: 72 larvae (4.5 ± 0.5 mm length) were separately placed in well plates, in a 12 : 12 LD regime. After 24 h, we added the synthetic DMD (Pherobees: Tribolur^®^). The larvae were exposed continuously to DMD throughout the experiment to imitate the practiced use of the pheromone traps. The plate was then placed under DanioVision camera, for 3 d under room temperature and light regime of 12‐h light (625 lux) and 12‐h dark, continuously tracking their movement and their activity (cm/10 min). As in most pheromone‐scented traps, we assumed that the synthetic DMD has a higher concentration (approximately × 10^6^) throughout the day than the daily peak of the naturally emitted DMD (633 ng per male daily) (Arnaud *et al.*, 2002).

**Adult–larva interactions**: 20 males, 20 females, and 40 larvae were each placed in separate 3.5 cm diameter arena. Adult arenas were positioned adjacent to the larval arena. To enable perception of vibrations and odor without physical contact, individual pairs of one larva and one adult were placed in larger, shared 9.5 cm diameter arenas. The arenas were placed for 3 d under the camera, under a 12‐h light/dark cycle to track their movement. As a control, 8 adults (4 males and 4 females) and 8 larvae were placed under the same conditions, with an empty, uncoupled 3.5 cm arena adjacent to their respective arenas.

**Movement tracking**: We analyzed activity levels and circadian rhythms of adults and larvae, characterized by peak activity time (“phase”) and rhythmicity (“period” length). The same activity parameters of larval and adult movement were tracked using Daniovision^®^ by Noldus under various conditions: exposure to conditioned versus new flour, a 12‐h light/dark cycle, and a constant darkness (free‐run) regime for 2 d. The beetle and larval movement in the interaction experiment was analyzed with EthoVision^®^. For further analyses, we averaged the distance moved every 10 min.

### Circadian genes expression pattern

We studied the oscillation in the expression of the clock genes (*Tctim* and *Tcper*) of *T. castaneum*’s larvae. Larvae were grouped in aggregations of 12, with a total of 108 individuals used: 84 for biological replicates and 24 for control replicates. Each larva was housed in a separate 2.5 mL Eppendorf tube with a breathing hole in the lid. To synchronize freezing, groups of 12 tubes were placed together in a metal net and subjected to simultaneous liquid nitrogen freezing. RNA was extracted from the larval heads collected and frozen every 4 h throughout the experiment. Following cDNA synthesis, quantitative real‐time PCR was used to analyze the daily expression of clock genes (details provided in the Materials and Methods section under Gene expression analysis).

**Gene expression analysis, total RNA extraction, and cDNA synthesis**: Total RNAs were isolated from samples using a modified manual extraction protocol with RiboEx^™^ solution (GeneAll, Germany). The final elution was performed in 40 μL DEPC‐treated water (twice at a volume of 20 μL) to ensure maximum recovery. Following RNA extraction, the samples were treated with gDNase and subsequently subjected to synthesis of first strand cDNAs from 1 μg of total RNA. gDNase treatment and cDNA synthesis were done using FastQuantRT Kit (TIANGEN, China).

**Primer design**: Based on the genome assembly Tcas5.2 (whole genome sequence of *Tribolium castaneum*, Ensemble Metazoa), we designed a set of primers to quantify the expression of the clock gene, *Tctim* (see Rath *et al.*, 2021) and *Tcper*. Primers were designed considering a length of 20–22 base pairs and GC content of 45%–55%. The SnapGene primer designing tool was used to evaluate the Tm and possibility of secondary structure formation of the designed primers. To make sure the primers were singletons, primer sequences were blast in ensemble genome of *Tribolium castaneum* for a single hit. Housekeeping gene primers for *ribosomal protein S3* (*rps3*) were used according to their description in the literature (Li *et al.*, 2018).

**Quantitative real time‐PCR**: Quantitative real‐time PCR (qPCR) was performed using 2× qPCR BIOSybrGreen Blue MixHi‐ROX (PCR Biosystems, UK) to quantify the expression of circadian genes. *Tribolium castaneum rps3* served as the reference gene for qPCR normalization. mRNA levels were expressed as relative expression using the ΔCt (delta cycle threshold) method, where ΔCt is the difference between the Ct (cycle threshold) values of the target gene and the reference gene. Normalized fold‐changes in target gene mRNA expression were calculated using the 2^−ΔΔCt^ method, where ΔΔCt is the difference between the ΔCt of the treated sample and the ΔCt of the control sample. Three biological replicates were used for each gene, with each replicate containing three technical repeats for result accuracy.

### Circadian activity pattern and statistical analysis

**Behavioral experiments**: All rhythmic activity was characterized using the nonparametric test JTK_cycle (Jonckheere‐Terpstra‐Kendall) algorithm in R (Hughes *et al.*, 2010). JTK_cycle is a nonparametric algorithm designed to detect rhythmic components in large datasets. Circadian activity is defined by a peak (“phase”) occurring every 24–28 h (“period,” i.e. Tau) with a statistical significance (*Q* < 0.05). The JTK_cycle identifies the time of the significant activity peak and the period length. The *Q*‐value was estimated using the Benjamini–Hochberg procedure (BH.Q) to control the false discovery rate due to multiple testing, for more details see Hughes *et al.* (2010). We used Fisher's exact test with a Bonferroni correction to compare the proportions of rhythmic and nonrhythmic individuals across treatments. To compare the effect of treatment on peak activity level, we employed a GLM model with light regime, treatment, and sex (where applicable) as factors. When evaluating the effect of conditioned flour, we used the Mann–Whitney *U* test.

Period length was evaluated under free run experiment (DD), as all others received LD conditions. This was compared to the 24‐h period of the LD (light‐dark) conditions using a Wilcoxon signed‐rank test.

We compared the effect of the treatment on activity level using GLM repeated measures models. Days served as the within‐subjects variable and individual beetle were treated as the between‐subjects variable. To estimate the overall effect of scotophase versus photophase, we pooled activity data from ZT 1 (Zeitgeber time 1, 1 h after turning the lights on) to ZT 12 (12‐h after turning the lights on), that is, the photophase, and ZT 13 to ZT 24, that is, the scotophase. Statistics were performed using the R (R Core Team, 2022). We used the “repeated” (Swihart *et al.*, 2023) package for the repeated measurement model.

**Gene expression**: For each replicate, the amplitude of each gene was calculated by dividing the maximum expression by the minimum. Circadian analysis was conducted using the JTK algorithm (Hughes *et al.*, 2010). Genes were classified as “cyclers” if they met two criteria: (1) a JTK *P*‐adjusted value less than 0.05 (indicating statistical significance) and (2) an amplitude greater than 1.5.

## Results

### Behavioral experiments

**Endogenous circadian clock**: The number of rhythmic individuals (defined by JTK statistics) did not differ under LD and DD conditions (*P* = 0.608). There was no significant difference between the period in DD condition from 24 h (*Z* = 51, *P* = 0.833). Under LD, 19 of 27 individuals were rhythmic with peak activity at 19.300 ± 5.352 ZT and under DD, 18 of 33 individuals were rhythmic with peak activity at 22.104 ± 7.873 ZT. Peak activity did not differ between treatments (GLM, *F*_(1,36)_ = 0.162, *P* = 0.689), and sex (female: 17.159 ± 0.895 ZT and male: 20.687 ± 1.326 ZT, GLM, *F*_(1,36)_ = 2.172, *P* = 0.129). Daily activity was also not effected by treatment (LD: 7.950 ± 0.053 cm/10 min and DD: 7.768 ± 0.053 cm/10 min, GLM, *F*_(1,108)_ = 0.534, *P* = 0.467) and sex (females: 8.200 ± 0.054 cm/10 min, males: 7.596 ± 0.050 cm/10 min, GLM, *F*_(1,108)_ = 2.235, *P* = 0.138).

**Fig. 1:**
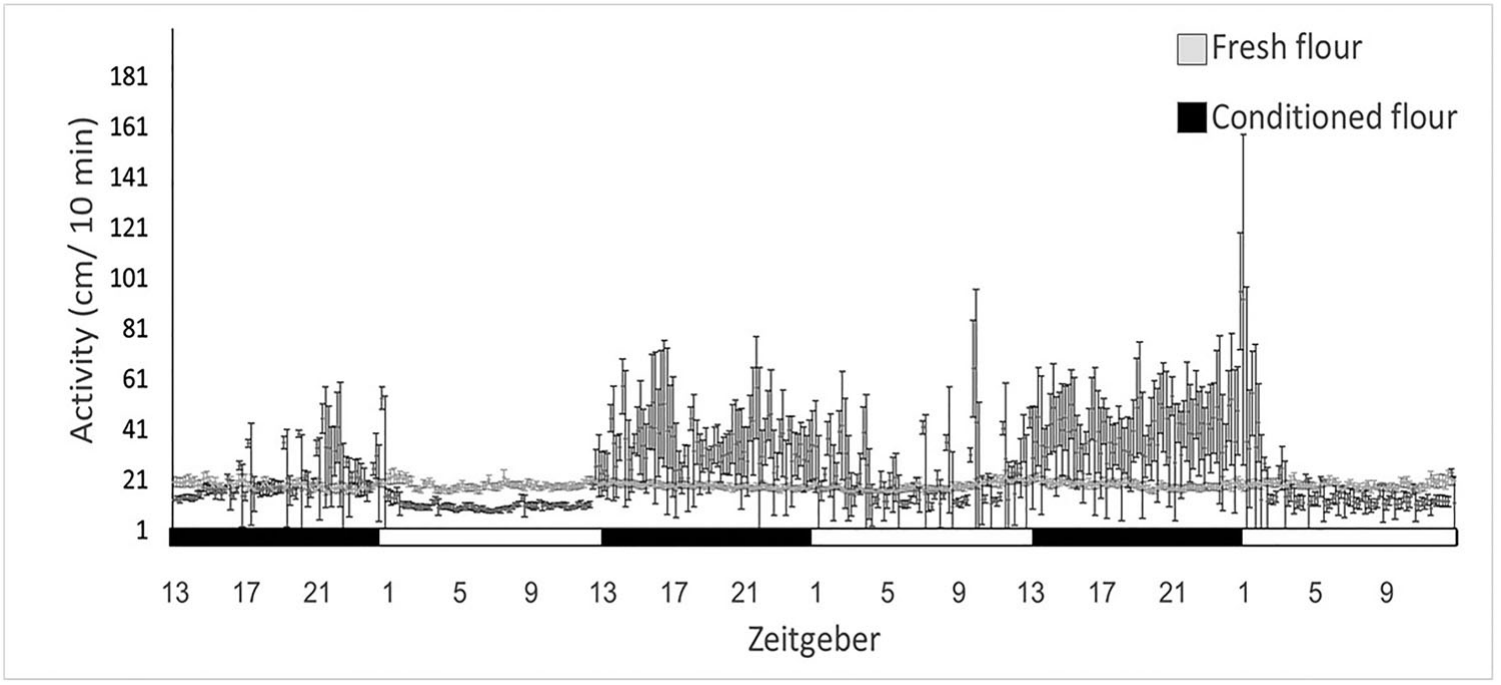
Activity level (mean ± SE) of *Tribolium castaneum*’s larvae with conditioned (black averaged line) and nonconditioned flour (grey averaged line), under 12‐h light and 12‐h day regime. White and black bars indicate light and dark phase, respectively. ZT 1 = 08:00, ZT 5 = 12:00, ZT 9 = 16:00, ZT 13 = 20:00. The number of rhythmic individuals was significantly higher in conditioned flour than nonconditioned flour (Fisher's exact test, defined by JTK statistics, *P* < 0.001).

**Conditioned flour**: The number of rhythmic individuals (defined by JTK statistics) was significantly higher in conditioned flour than nonconditioned flour (Fig. 1, 22 of 24 and 11 of 22 individuals, respectively, *P* < 0.001). Mean activity peak in the conditioned flour occurred at the scotophase (18.948 ± 1.168 ZT) and beetles in the nonconditioned flour mean activity peak occurred at the photophase (4.352 ± 8.952 ZT). The variation in the peak of activity was 12 times larger in the nonconditioned flour resulting in no significant difference in peak activity time (*Z* = −1.758, *P* = 0.091). Daily activity level was not affected by the treatment (conditioned: 24.744 ± 3.568 ZT and fresh: 18.806 ± 0.488 ZT, GLM, *F*_(1,272)_ = 2.631, *P* = 0.106). There was a significant effect of phase (photophase: 18.352 ± 1.642 ZT and scotophase: 25.456 ± 3.367 ZT, GLM, *F*_(1,272)_ = 4.140, *P* < 0.05), and the interaction between phase and flour condition (GLM, *F*_(1,272)_ = 4.063, *P* < 0.05). The switch between scotophase to photophase increased activity significantly more in conditioned flour than fresh flour (conditioned flour: scotophase: 31.525 ± 6.355, photophase: 17.963 ± 3.101; fresh flour: scotophase 18.835 ± 0.737, photophase 18.776 ± 0.646).

**Fig. 2:**
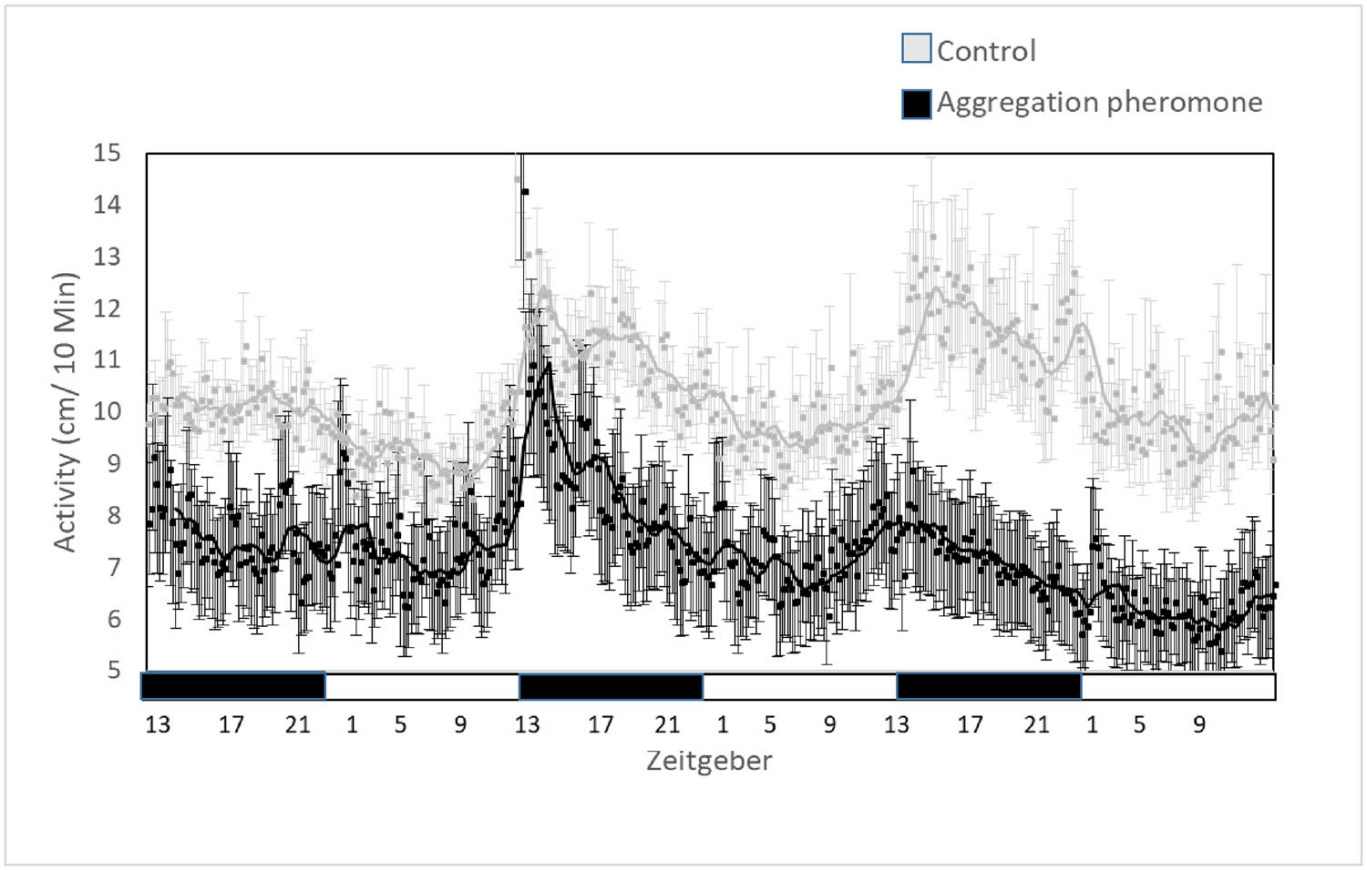
Activity level (mean ± SE) of *Tribolium castaneum*’s larvae (mean ± SE) under 12‐h light and 12‐h day regime with (black averaged line) and without (grey averaged line) pheromone DMD. White and black bars indicate light and dark phase, respectively. ZT 1 = 08:00, ZT 5 = 12:00, ZT 9 = 16:00, ZT 13 = 20:00. DMD treatment significantly reduced larvae activity (GLM, *F*_(1,411)_ = 84.563, *P* < 0.0001).

**Exposure to DMD**: Number of rhythmic individuals (defined by JTK statistics) did not differ between treated and nontreated individuals (10 of 36 and 7 of 36, *P* = 0.165). DMD did not shift the activity peak (control: 18.978 ± 1.198 ZT and DMD: 18.500 ± 1.039 ZT, *Z* = −0.381, *P* = 0.703). DMD treatment however reduced larvae activity (control: 10.331 ± 0.273 cm/10 min, DMD: 7.314 ± 0.184 cm/10 min, GLM, *F*_(1,411)_ = 84.563, *P* < 0.0001, Fig. 2). Larvae activity was also affected by phase (photophase: 8.032 ± 0.223 cm/10 min and scotophase: 8.958 ± 0.255 cm/10 min, GLM, *F*_(1,410)_ = 8.571, *P* < 0.010) with no interaction between phase and DMD treatment (GLM, *F*_(2,410)_ = 0.219, *P* = 0.640).

**Fig. 3:**
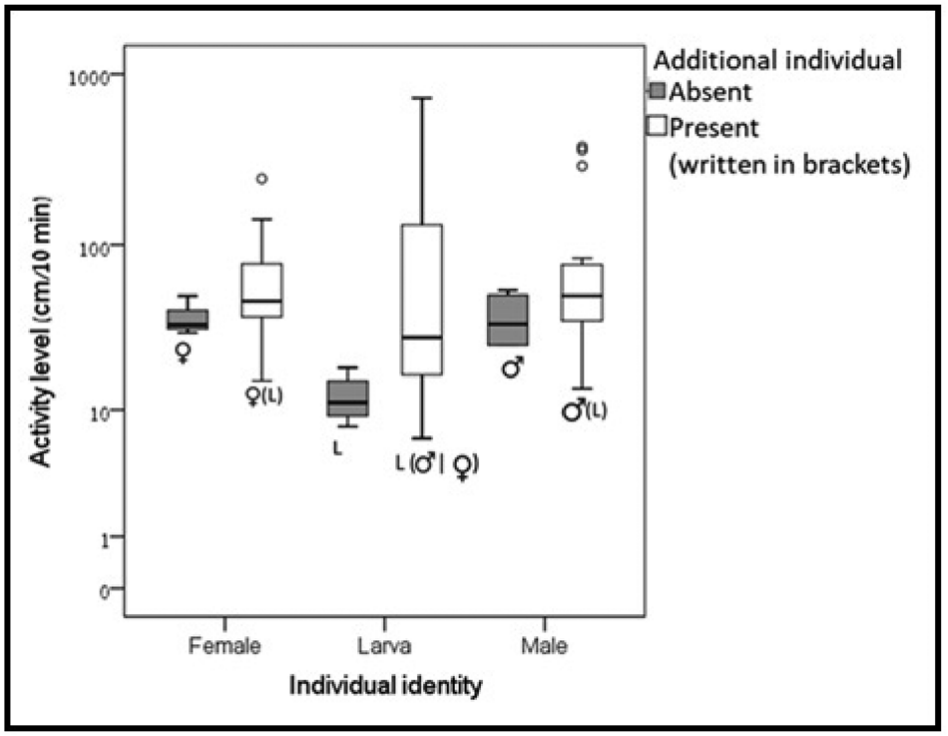
Activity level of females, larvae, and males interacting with an additional individual (larvae‐male/female or male‐larva, female‐larva). Each box plot represents the median for the same 10 experimental colonies and 25th and 75th percentiles. Whiskers depict the values within 1.5 times the interquartile range. Extreme outliers are denoted by circles. The activity level was significantly higher with the presence of an additional individual in the arena (GLM, *F*_(1,473)_ = 11.742, *P* < 0.001).

**Adult larvae interactions**: There was no difference in the frequency of pairs with two rhythmic beetles compared to the sum of pairs with one or two arrhythmic beetles (20 of 32 and 12 of 32 respectively, *P* = 0.079) and between adults and larvae (28 of 39 and 34 of 40, *P* = 0.734). Peak activity time was only affected by the individual's sexual maturity (female: 14.730 ± 0.651 ZT, male: 14.130 ± 0.461 ZT, Larva:10.660 ± 0.652 ZT, *F*_(1,114)_ = 4.578, *P* < 0.05). A *post hoc* test indicated a significant difference between adults and larvae (Wilcoxon signed‐rank test, male‐larva: *Z* = 1.433, *P* = 0.152, female‐larva: *Z* = −2.012, *P* < 0.05) and neither an effect of an additional individual in the arena (alone: 12.29 ± 0.889 ZT, pair: 12.620 ± 0.443 ZT, *F*_(1,114)_ = 0.106, *P* = 0.745) nor of the experimental day (Day 1: 13.040 ± 10.454 ZT, Day 2: 12.240 ± 0.657 ZT, Day 3: 12.400 ± 0.694 ZT, *F*_(1,114)_ = 4.578, *P* = 0.984). The activity level was affected by the additional individual in the arena (alone: 29.732 ± 2.179 cm/10 min, pair: 66.656 ± 7.687 cm/10 min, *F*_(1,473)_ = 11.742, *P* < 0.001) (Fig. 3) and not by the experimental day (Day 1: 53.196 ± 10.454 cm/10 min, Day 2: 61.607 ± 11.055 cm/10 min, Day 3: 63.013 ± 10.750 cm/10 min, *F*_(1,473)_ = 0.016, *P* = 0.984) or the individual's sexual maturity (female: 51.382 ± 4.373 cm/10 min, male: 69.924 ± 69.924 cm/10 min, larva: 57.891 ± 10.927 cm/10 min, *F*_(1,473)_ = 0.567, *P* = 0.568) or phase (scotophase: 63.197 ± 10.565 cm/10 min, photophase: 55.342 ± 6.507 cm/10 min, *F*_(1,473)_ = 0.287, *P* = 0.592).

### Circadian genes expression pattern

**Fig. 4:**
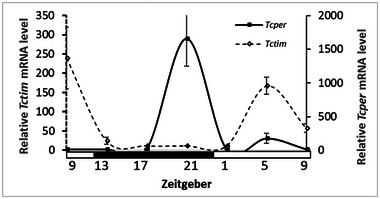
*Tribolium castaneum*’s *Tctim* (fractured line) and *Tcper* (solid line) mRNA expression (mean ± SE) in the larvae under 12‐h light and 12‐h day regime. White and black bars indicate light and dark phase, respectively. ZT 1 = 08:00, ZT 5 = 12:00, ZT 9 = 16:00, ZT 13 = 20:00. The expression of Tctim and Tcper by the larvae was rhythmic with a significant peak (JTK, *P* < 0.0001). While *Tctim* expression is during the photophase *Tcper* expressed during scotophase and in much higher levels.

The expression of *Tctim* and *Tcper* by the larvae was rhythmic with a significant peak (JTK, *P* < 0.0001). *Tctim* peaks at Zeitgeber 0 (lights on 7:00 AM, JTK, *P* < 0.0001 with an amplitude of 93.022, *n* = 12) and *Tcper* peaks at Zeitgeber 12 (lights of 19:00 PM, JTK, *P* < 0.0001 with an amplitude of 12.440, *n* = 12, Fig. 4).

## Discussion

This study is among the few to investigate the circadian system in insect larvae. We confirm the presence of an endogenous circadian rhythm in *Tribolium castaneum* larvae. Contrary to our initial hypothesis, larval peak activity consistently occurred during the scotophase (dark), regardless of exposure to aggregation cues: presence of DMD, conditioned flour, or adults’ presence. Interestingly, the absence of aggregation cues with abundance of unconditioned flour led to a substantial decrease in the proportion of rhythmically active larvae. These results clearly indicate that social interactions, rather than light, induce rhythmicity in the gregarious *T*. *castaneum* larvae.

*Tribolium castaneum* larvae showed a nocturnal rhythmic activity pattern that switches to daily activity in the adults. The origin of rhythmicity changes between insect larvae and adults is a subject of debate: Is it driven by changes in the endogenous clock or downstream pathways (Uryu & Tomioka, 2014). The study on *Tribolium* beetles offers valuable insights. *Tribolium* larvae peak activity aligns with the expression patterns of their clock genes: *Tctim* peaks during the day (photophase), while *Tcper* peaks at night (scotophase). Interestingly, this clock undergoes a significant shift during development. *Tribolium* adults shift to diurnal activity that coincides with a mirror‐image change in gene expression: *Tctim* peaks at night, while *Tcper* peaks during the day (Rath *et al.*, 2021). This finding suggests that the change in activity pattern is indeed driven by modifications within the endogenous clock itself, not just downstream pathways. However, this finding contrasts with other studied insects like the cricket *Gryllus bimaculatus* (Uryu & Tomioka, 2014). Contrary to *Tribolium*, crickets switch from diurnal activity as nymphs to nocturnal activity as adults, yet their clock genes maintain the same expression pattern (Uryu & Tomioka, 2014). It is also a contrast to a recent research on strains of *T*. *castaneum* demonstrating nocturnal activity, though similarly to our research showing rhythmicity (Reshma *et al.*, 2024). One important difference between their research and ours is light intensity. Ours was at least 6 times more intense making it an important future direction to examine whether light intensity can modify the activity patterns and cause a phase shift. Furthermore, it would be interesting to check the gene expression under conditioned flour. Finally, it is worthwhile to examine whether other Zeitgebers, such as stress from adult scent, also modify the genes.

Interspecific differences in eye development, and their resulting impact on light sensitivity, may explain these observed variations. Species with significant developmental changes in eye structure and light perception (like *Tribolium*) might be more likely to exhibit endogenous clock shifts, while those with consistent light sensitivity (like crickets) might rely more on downstream adaptations. The eyes in most insects are the only organ receiving photic information necessary for the entrainment of the endogenous circadian clock (Tomioka & Matsumoto, 2019). Developmental changes in eye structure can affect how light signals reach the photoreceptors. This in turn can play a crucial role in the entrainment of the endogenous clock (Tomioka & Matsumoto, 2019). Holometabolous insects, for example, *T. castaneum*, have two different eye structures within their development (Tomioka & Matsumoto, 2010). Eyes in the adults consist of ommatidia, containing hundreds of specialized photoreceptor cells as opposed to the smaller clusters of cells called stemmata in the larvae (Hunter‐Ensor *et al.*, 1996). Consequently, changes in the endogenous clock can lead to different behavioral patterns. However, in hemimetabolous insects, for example, crickets, nymphal eye develops into the adult eye (Friedrich, 2006). Thus, the endogenous system characteristics are maintained and any adaptation to optimal activity time may occur via downstream pathways. Future studies should investigate the ontogeny of endogenous clock regulation between hemimetabolous and holometabolous insects to clarify the relative roles of the endogenous clock and downstream pathways in shaping circadian rhythms.

While light and temperature are recognized as the main forces synchronizing internal clocks across various organisms (Dunlap *et al.*, 2004), survival in the wild necessitates that animals adapt their behavior to social cues as well (Regal & Connolly, 1980; Levine *et al.*, 2002; Mistlberger & Skene, 2004; Fuchikawa *et al.*, 2016; Fujioka *et al.*, 2019; Rath *et al.*, 2021; Siehler *et al.*, 2021). Strong evolutionary pressures like mating harassment, predation, or cannibalism may temporally limit activity to specific times to minimize risk. Segregation together with synchronizing activity with cohorts may have a diluting effect; a unified activity time decreases the chance of any single individual being targeted. In environments with lower risk, the benefit from synchronized activity for diluting predation risk becomes less crucial, potentially leading to more diverse individual activity patterns.

Recent studies suggest that social cues indicating potential sexual harassment might influence daily activity patterns in *T. castaneum* (Rath *et al.*, 2021; Benita *et al.*, 2024). We predicted that the impact of social cues on the circadian clock might be important also in avoiding cannibalism at the larvae developmental stage (Stevens, 1989). Social synchronization of circadian rhythms can enable *T. castaneum* larvae to segregate their activity from cannibalistic adult's presence. In the current study, however, when cues indicated potential cannibalistic risk (low DMD, presence of adult, and conditioned flour), larval activity increased, suggesting possible dispersal behavior to avoid danger. This contrasts with expectations of reduced activity and highlights the sensitivity of larvae to social cues and their adaptive response to potential threats. Nevertheless, constant cues release (exposure to DMD and conditioned flour) and rhythmic cues release (the presence of another individual) did not shift individual's peak activity time and there was a segregation in activity time between larvae and adults under all conditions. Notably, adults remained active during the photophase and larvae at the scotophase. Remarkable reduction of this robust pattern occurred when placing larvae in fresh flour, that is, no social cues indicating on decrease in cannibalism risk. Under this experimental setup, the number of rhythmic individuals significantly decreased and variability of peak activity time increased. Thus, our study supports previous studies (Loe *et al.*, 2007; Bonnot *et al.*, 2016) suggesting that when the environment is free of predators, that is, in the current study unconditioned flour, there is the possibility of extended period of activity, that is, lower number of rhythmic individuals and higher variation between individuals in the peak activity. This extended activity could potentially increase food consumption, leading to larger and possibly fitter adults. Future research could explore the exact mechanisms behind this extended activity and its potential fitness benefits in a predator‐free environment.

The critical role of social cues in shaping synchronized rhythms, even under constant light conditions, suggests that social environment may supersede photic entrainment of the endogenous clock for *T. castaneum*. This finding is supported by Reshma *et al.* (2024) documenting that adult beetles exposed to constant light conditions (LL, DD) maintained rhythmicity. This further aligns with studies on honeybees (Fuchikawa *et al.*, 2016; Siehler *et al.*, 2021) and *Drosophila* (Vanin *et al.*, 2012), suggesting that social cues can supersede photic entrainment under ecologically relevant conditions. Future studies should consider cues released by parasitoids wasps which could also influence flour beetle rhythms. Larvae are potential hosts for the parasitoids *Cephalonomia tarsalis* (Hymenoptera; Bethylidae) and *Holepyris sylvanidis*, both exhibit diurnal activity (personal observation DG). *H. sylvanidis* uses the cuticular hydrocarbons of *Tribolium* sp. larvae for both trail following and host recognition (Fürstenau & Hilker, 2017). Intriguingly, parasitoids spend significantly more time on freshly laid CHCs’ trails than on 24 h ones (Awater‐Salendo *et al.*, 2021). Thus, by being active at night, larvae may be able to avoid being detected by the daily parasitoids and parasitized by these insects.

## Conclusions

This study contributes to the limited research on the ontogeny of insect circadian system (Uryu & Tomioka, 2014; Tomioka & Matsumoto, 2015; Niepoth *et al.*, 2018). The study highlights *Tribolium* larvae's robust endogenous circadian rhythm, likely adapted to life‐stage‐specific environmental pressures, like cannibalism and predation. The evident switch in peak expression and peak activity time highlights the ongoing debate: do changes in the circadian gene oscillator itself, or downstream effectors, primarily drive shifts in behavioral rhythmicity?

Furthermore, our study suggests the supremacy of social environment over photic entrainment and indicates that to understand how the circadian clock organizes behavior and its functional significance it is imperative to study animals under their specific natural context. Future studies should pinpoint the exact life stage where the switch in peak expression occurs. On a practical manner, despite the common presence of parasitoid wasps in storage facilities, the wasps are regarded as a “poor” biocontrol agent (Harush *et al.*, 2021). Understanding *Tribolium*’s responses to stimuli like light or odors indicating on cannibalism risk or parasitoids wasp can assist in the development of traps, repellents, or attractants that strategically exploit the pest's behavior for more effective control.

## Disclosure

The authors declare no conflict of interest.
